# From low- to high-level resistance: dynamic transcriptional adaptations and efflux pump amplification in cefiderocol-stressed *Pseudomonas aeruginosa*

**DOI:** 10.3389/fmicb.2026.1871230

**Published:** 2026-08-13

**Authors:** Ting Zhang, Hanxu Hong, Jun Wang, Yuxuan Liu, Yun Cao, Yang Liu, Wei Zhang

**Affiliations:** 1 Department of Respiratory and Critical Care Medicine, The First Affiliated Hospital, Jiangxi Medical College, Nanchang University, Nanchang, China; 2 Department II of Respiratory and Critical Care Medicine, Jiangxi Provincial People’s Hospital, The First Affiliated Hospital of Nanchang Medical College, Nanchang, China; 3 JXHC Key Laboratory of Respiratory Diseases, Jiangxi Provincial People’s Hospital, The First Affiliated Hospital of Nanchang Medical College, Nanchang, China; 4 Department of Clinical Laboratory, The First Affiliated Hospital, Jiangxi Medical College, Nanchang University, Nanchang, China; 5 School of Public Health, Jiangxi Medical College, Nanchang University, Nanchang, China; 6 Jiangxi Medical Center for Critical Public Health Events, The First Affiliated Hospital, Jiangxi Medical College, Nanchang University, Nanchang, China

**Keywords:** cefiderocol, efflux pump, fitness cost, *Pseudomonas aeruginosa*, resistance, transcriptomics

## Abstract

*Pseudomonas aeruginosa* is a bacterium with high antimicrobial resistance to several drugs including carbapenems. Recently, resistance to cefiderocol (FDC), a new siderophore cephalosporin, has been reported despite its rare use in clinical practice. Therefore, this study aimed to investigate the dynamics of different resistance mechanisms in *P. aeruginosa* that evolve under FDC pressure, as well as the interactions between these mechanisms in evolutionary trajectories to guide clinical medication. *P. aeruginosa* ATCC 27853 was continuously induced with subinhibitory concentrations of cefiderocol *in vitro* to generate strains with different resistance levels. Phenotypic adaptability and molecular regulatory networks during the evolution of resistance were systematically elucidated using growth curves, pairwise competition assays, transcriptomics, RT-qPCR and efflux inhibition assays. The minimum inhibitory concentration (MIC) of the induced FDC-resistant strain increased from 0.125 to 64 μg/mL. Simultaneously, the growth rate and peak declined below those of the parental strain. The pairwise competition assay showed that the relative fitness of resistant strains versus parental strains was < 1 in LB and ID-CAMHB broths. Transcriptome analysis revealed that the MIC of the induced FDC-resistant strain was related to the dose of efflux pumps, together with a layer-by-layer regulation of gene expression, to adapt to environmental stress. In the initial resistant strain, efflux pumps were slightly upregulated, and energy metabolism was downregulated. Conversely, in the strain with a MIC of 64 μg/mL, efflux pumps were significantly up-regulated and drove bacterial reprogramming to induce resistance, particularly the RND efflux pump component oprM, along with multiple ABC transporters. RT-qPCR validated the significant upregulation of oprM, opuC, and opuBD (*p* < 0.05), confirming their central role. Furthermore, in the presence of the efflux pump inhibitor phenyl-arginine β-naphthylamide (PAβN), the resistant strains exhibited significantly reduced MICs for FDC. In conclusion, these data indicate that multiple mechanisms of action are involved in the antibacterial activity of *P. aeruginosa* against FDC. Notably, the evolution of resistance was associated with the dose-dependent upregulation of core efflux pumps, complemented by stage-specific global physiological remodeling; however, it also came at a fitness cost.

## 1 Introduction

Studies have increasingly reported that *Pseudomonas aeruginosa*, an important opportunistic pathogen in hospital-acquired infections, exhibits resistance to several antibiotics, with particularly increasing resistance rates to carbapenems. *P. aeruginosa* is one of the three bacterial species listed for critical priority research by the World Health Organization (Elfadadny et al., 2024; Tacconelli et al., 2018). Additionally, this bacterium has complex resistance mechanisms, which involve reduced outer membrane permeability, overexpression of efflux systems, and production of antibiotic-inactivating enzymes. Particularly, the efflux pump system plays a key role in multiple drug resistance (Sastre-Femenia et al., 2025; Serepa-Dlamini et al., 2025). Therefore, new antibiotics to treat these infections are urgently required.

Cefiderocol (FDC), a novel siderophore cephalosporin, enters cells via the bacterial iron uptake system through a “Trojan horse” mechanism (Wang et al., 2024). FDC has demonstrated excellent activity against multiple drug-resistant gram-negative bacteria, including *P. aeruginosa* (Karakonstantis et al., 2024; Yan et al., 2025). However, recent studies have reported the emergence of FDC resistance in clinical isolates from China, where FDC was launched on January 8th this year, and the resistance mechanisms remain unclear (Hong et al., 2024; Sastre-Femenia et al., 2025). This finding highlights the importance of understanding the evolutionary pathways of *P. aeruginosa* resistance to FDC.

In this study, we induced *P. aeruginosa* ATCC 27853 with sub-inhibitory concentrations of FDC *in vitro* to construct strains with different resistance levels. We systematically elucidated the phenotypic fitness and molecular regulatory networks during the evolution of resistance, providing a theoretical basis for the clinical application of FDC and combination therapy strategies. This study is fundamental for informing antimicrobial stewardship and designing strategies to mitigate resistance development.

## 2 Materials and methods

### 2.1 Bacterial strains, and antimicrobial susceptibility testing

*P. aeruginosa* ATCC 27853 was inoculated onto Luria-Bertani (LB, Becton Dickinson and Co., United States) agar plates overnight at 37°C. The Antimicrobial Susceptibility Test (AST) was performed using the VITEK-2 system (bioMérieux, Lyon, France) and the results were verified in our laboratory using the broth microdilution method. The minimum inhibitory concentration (MIC) of FDC was determined to be 0.125 μg/mL using iron-deficient cation-adjusted Mueller-Hinton broth (ID-CAMHB) according to the Clinical and Laboratory Standards Institute (CLSI) guidelines (CLSI, 2023). Each isolate was independently tested three times and the results were interpreted according to the guidelines.

### 2.2 *In vitro* progressive FDC exposure

The progressive antibiotic exposure experiment was initiated with *P. aeruginosa* ATCC 27853 (initial MIC = 0.125 μg/mL) as the parental strain, following previously established methods (Galdino et al., 2024; Liu et al., 2023). Bacteria were serially passaged in ID-CAMHB broth containing FDC at a dynamic sub-inhibitory concentration (1/2 of the current MIC) and the MIC was measured daily. The antibiotic concentration was increased only when an increase in the MIC was observed. Induced strains were preserved at −80°. Strains with MICs of 4 μg/mL and 64 μg/mL were designated PA27853MIC4 and PA27853MIC64, respectively, and selected for subsequent experiments.

### 2.3 Whole genome sequencing and analysis

Whole genome sequencing (WGS) was performed on the Illumina HiSeq X-ten platform with a read length of 150 bp. The genomic DNA of PA27853MIC4 and PA27853MIC64 were extracted using the TIAN amp Bacteria DNA Kit (DP302, Tiangen Biochemical Technology [Beijing] Co., Ltd.) following the manufacturer’s instructions. Clean reads were mapped to the reference *P. aeruginosa* ATCC 27853 genome (NCBI Reference Sequence: NZ_CP101912.1, Assembly GCF_024507955.) using Bowtie 2 (Langmead and Salzberg, 2012). Variant calling was performed using the Samtools (Li et al., 2009) and GATK (McKenna et al., 2010). Variants with a sequencing depth > 10-fold and a mutation frequency > 0.8 were identified as the final confident loci.

### 2.4 Fitness cost

#### 2.4.1 Growth curve measurements

Single colonies of *P. aeruginosa* ATCC 27853 and the induced strains were inoculated into LB and ID-CAMHB broths placed in a steady temperature shaking incubator at 37°C until the logarithmic growth phase (approximately 1 × 10^9^ CFU/mL). The initial OD_600_ densities of the bacterial suspensions were adjusted to ensure consistency. Subsequently, 100 μL of the bacterial suspension was transferred to 100 mL of LB broth (or ID-CAMHB broth) and incubated at 37°C with shaking. Samples of 100 μL were collected every hour in a sterile environment to measure the OD_600_ value using a spectrophotometer and the results were recorded. Bacterial growth was monitored by measuring OD_600_ over time. The resulting kinetics were presented as growth curves.

#### 2.4.2 Pairwise competition assay

The parental *P. aeruginosa* strain ATCC 27853 and the induced strain PA27853MIC64 were cultured overnight to a 0.5 McFarland standard. Equal volumes of 50 μL from each strain were mixed in 5 mL of LB and ID-CAMHB broths, and recorded as d0. The mixed bacterial suspension was incubated at 37°C with shaking for 72 h. Every 24 h, 5 μL cultures were reinoculated in 5 mL of fresh broth, recorded as d1, d2, d3. The mixed cultures from d0–d3 were subjected to a series of 10-fold dilutions and plated on LB agar plates with or without 4 μg/mL FDC. Three parallel groups were used for each experiment, and the names of these groups were A, B, and C. The relative competition index was calculated as W = ln(NRt/NR0)/ln(NSt/NS0). NR is the number of resistant clones and NS is the number of susceptible clones, with values < 1 indicating the fitness cost.

### 2.5 Total RNA isolation and RNA sequencing

RNA sequencing of *P. aeruginosa* parental and induced strains was performed to further clarify the regulatory effect of FDC on *P. aeruginosa* gene expression. Each experiment was repeated three times. Cryopreserved *P. aeruginosa* ATCC27853, PA27853MIC4, and PA27853MIC64 were activated and cultured until the logarithmic growth phase. Bacterial cells in the logarithmic growth phase were harvested by centrifugation to obtain cell pellets. Total RNA was extracted using the TRIzol reagent. The integrity and quality of the total RNA were assessed using an Agilent Bioanalyzer (Agilent Technologies, United States), followed by ribosomal RNA (rRNA) depletion using the Illumina Ribo-Zero Plus rRNA Depletion Kit (Illumina, United States). Purified messenger RNA (mRNA) was used for RNA-Seq library construction in accordance with the manufacturer’s instructions for the KAPA Stranded RNA-Seq Library Preparation Kit for Illumina Platforms (KAPA Biosystems, United States). Libraries were sequenced on an Illumina NovaSeq 6000 platform (Illumina, United States) to generate 150-bp paired-end reads. Raw reads were assessed for quality, followed by adapter trimming using the fastp pipeline (v0.21.0) (Chen et al., 2018). Clean reads were aligned to the genome of *P. aeruginosa* ATCC27853 using Bowtie2 (v2.3.4.1) (Langmead and Salzberg, 2012).

### 2.6 Bioinformatics analysis of the differentially expressed genes

Only genes with a minimum of 50 mapped reads from at least three samples across the two experimental groups were retained for downstream analysis. Among them, differentially expressed genes (DEGs) were identified using the RUVSeq package (Risso et al., 2014) with the following screening criteria: a minimum expression level of 20 Reads Per Kilobase per million mapped reads (RPKM) in at least one group, an adjusted *P*-value (*q*-value) < 0.01 after Benjamini–Hochberg multiple test correction, and an absolute fold change (| FC|) > 1.5. A volcano map of DEGs was drawn for subsequent analysis, with a focus on analyzing the correlation between the amplitude of gene expression change (log2FC) and MIC.

### 2.7 Gene Ontology and Kyoto Encyclopedia of genes and genomes database pathway enrichment analysis

Differential gene enrichment analysis included Gene Ontology (GO) and Kyoto Encyclopedia of Genes and Genomes database (KEGG) analyses. GO terms were annotated using the InterProScan software (Jones et al., 2014), and gene functions were annotated using the KEGG database.^1^ The analyses were performed using the enriched function from the R package clusterProfiler (Xu S. et al., 2024). Enrichment plots were generated using the ggplot2 package. Heatmaps of the DEGs were constructed using the R package, pheatmap.

### 2.8 qRT-PCR experiments

Real-time quantitative reverse transcription PCR was used to detect the expression levels of six key efflux pump genes (*oprM, lolC_E_F, modB, opuA, opuC, and opuBD*) in the ATCC27853, PA27853MIC4 and PA27853MIC64 groups. The mean expression of the strains in the ATCC27853 group was used as the reference value. The expression level was assessed using TB Green Premix Ex Taq (Takara, Kyoto, Japan) in a LightCycler 480 system (Roche, Rotkreuz, Switzerland), with triplicate samples for each isolate, replicated thrice independently using the comparative CT method. 2^–ΔΔCT^ method was used for relative quantitation of the six resistance genes. The mean CT values from triplicate runs were used as input data (the CT value range for all triplicates was < 0.5).

### 2.9 Efflux inhibition assay

P. *aeruginosa* ATCC 27853, PA27853MIC4, and PA27853MIC64 were cultured in ID-CAMHB. The impact of 25 μg/mL efflux pump inhibitor phenyl-arginine β-naphthylamide (PAβN) on MIC values was quantified by the micro-broth dilution assay according to a previously described method (Gbian and Omri, 2021). MIC decreases of two-fold or more in combination with PAβN were regarded as a positive efflux effect.

### 2.10 Statistical analysis

Statistical analyses of data were expressed as mean ± standard error. Independent *t*-tests were used for comparisons between two groups, and one-way ANOVA was used for comparisons between more than two groups. All statistical analyses were performed using GraphPad Prism 9.1.0 (GraphPad Software Inc., San Diego, CA, United States). All statistical tests were two-sided and *p* < 0.05 was considered statistically significant.

## 3 Results

### 3.1 MIC variation patterns of induced *P. aeruginosa* ATCC 27853

**FIGURE 1:**
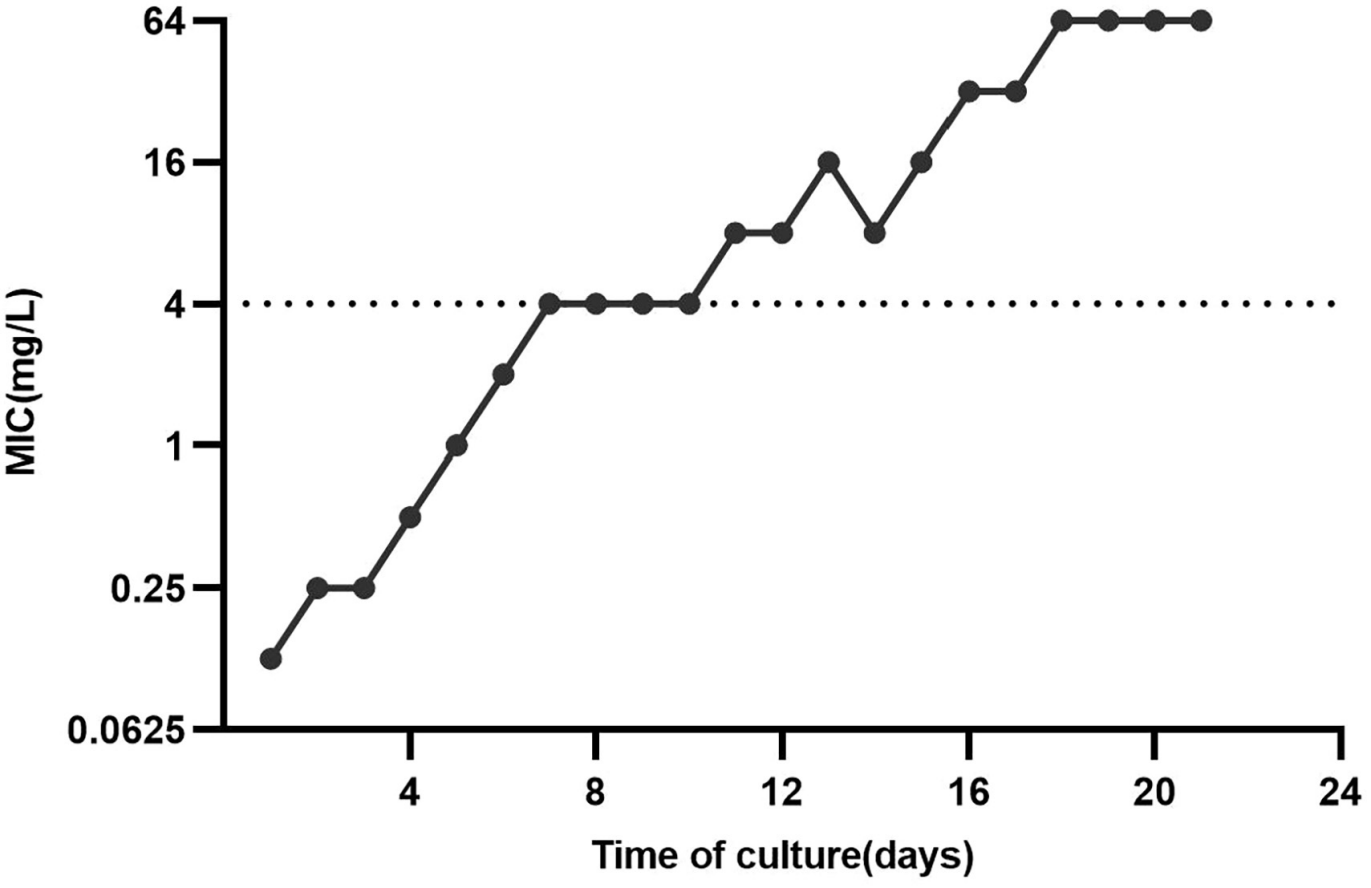
Course of progressive exposure of *P. aeruginosa* to sub-MIC FDC concentrations.

After 1 week of treatment with FDC, *P. aeruginosa* ATCC 27853 had an FDC MIC of 4 μg/mL. The MIC increased slowly with the induction time (Figure 1), and resulted in resistant strains with different MICs after several rounds of passaging. Based on the CLSI resistance breakpoint (4 μg/mL) and the maximum achieved MIC (64 μg/mL), these strains were selected for subsequent experiments, labeled as PA27853MIC4 and PA27853MIC64, respectively.

### 3.2 FDC resistance pays fitness costs

#### 3.2.1 Growth curve analysis

**FIGURE 2:**
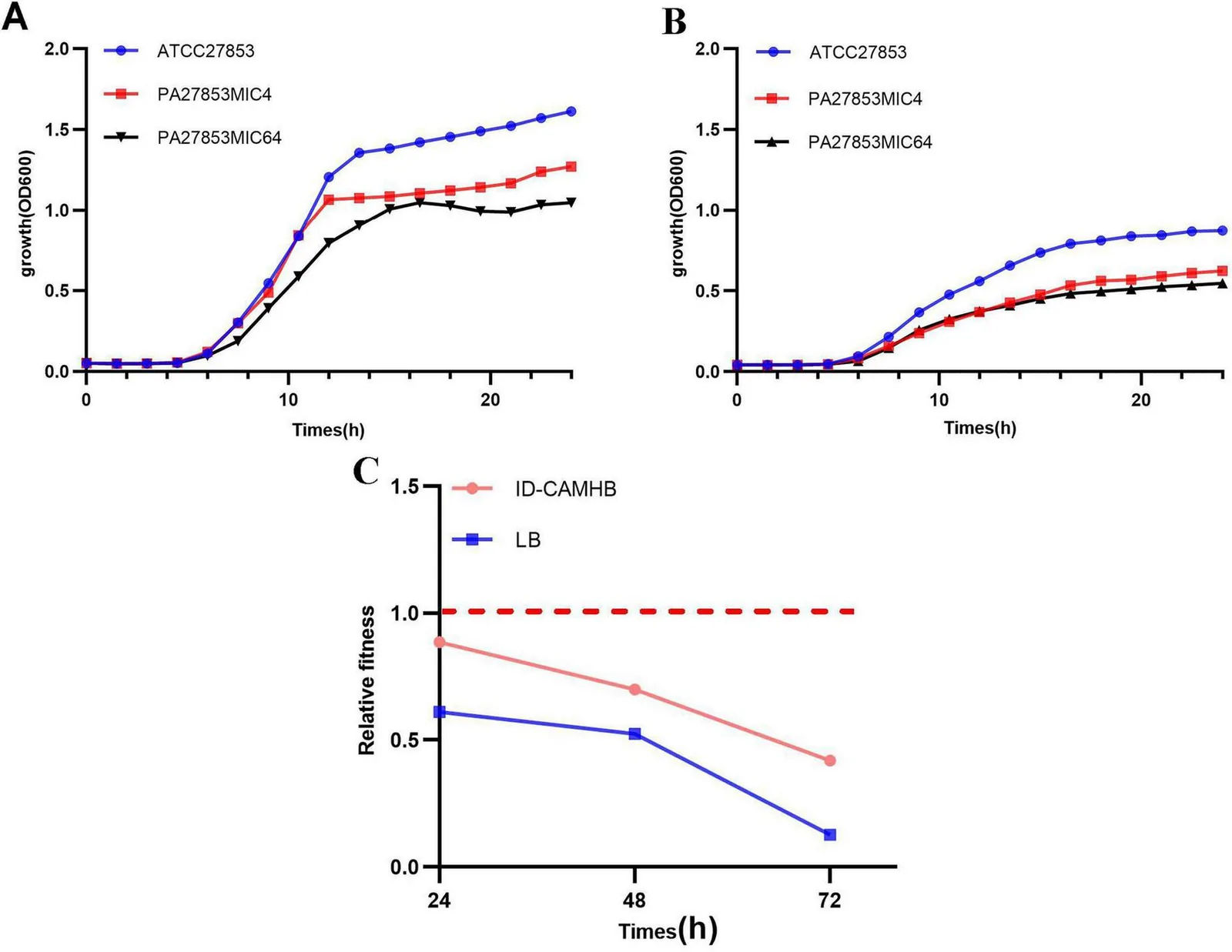
Fitness cost analysis. **(A)** The growth curves of induced strains and parental strains in LB broth. **(B)** The growth curves of induced strains and parental strains in ID-CAMHB broth. **(C)** The pairwise competition of induced strains and parental strains in LB broth and ID-CAMHB broth.

In LB and ID-CAMHB broths at 37°C, the induced resistant strains grew slower than the parental *P. aeruginosa* ATCC 27853 strain, reaching a lower and later plateau. Particularly, PA27853MIC64 exhibited the slowest growth, lowest peak, and the latest plateau (Figures 2A,B). This inverse correlation between increased induced MIC and reduced growth indicates a fitness cost associated with FDC resistance.

#### 3.2.2 Pairwise competition experiment

PA27853MIC64 and *P. aeruginosa* ATCC 27853 were subjected to a fitness cost evaluation to further investigate the relationship between FDC resistance and fitness. The pairwise competition experiment showed that under LB and ID-CAMHB broths culture conditions, the RF values of the induced resistant strains and parental strains were < 1, indicating a certain fitness cost, with a greater fitness cost in LB than in ID-CAMHB (Figure 2C).

### 3.3 Effects of FDC on the transcriptomic profile

#### 3.3.1 Bioinformatics analysis of the differentially expressed genes

**FIGURE 3:**
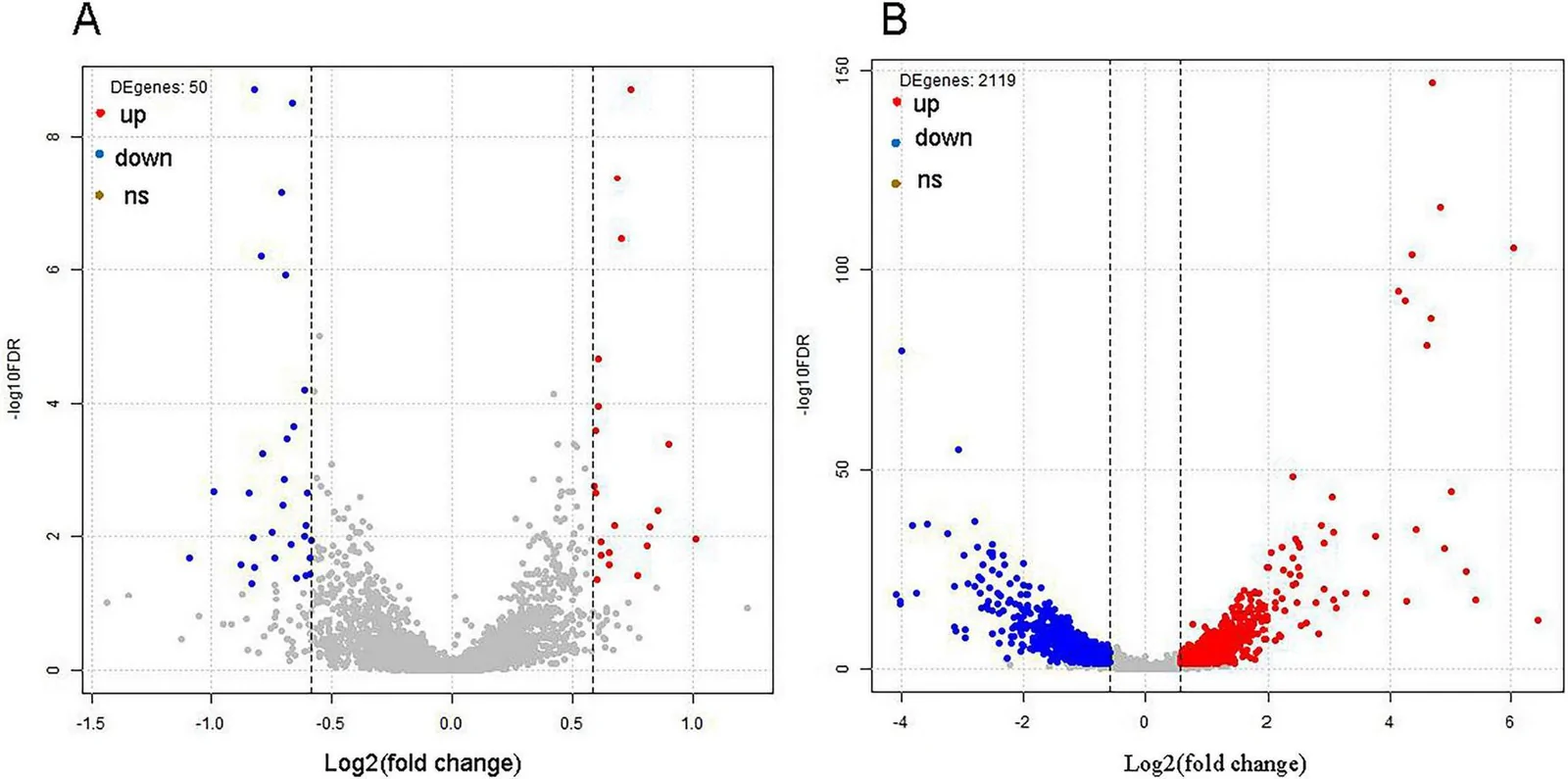
Transcriptomic changes in PA27853MIC4 and PA27853MIC64. **(A)** Volcano plot of differentially expressed genes (DEGs) identified by RNA-Seq in PA27853MIC4 compared to *P. aeruginosa* ATCC27853. **(B)** Volcano plot of DEGs identified by RNA-Seq in PA27853MIC64 compared to *P. aeruginosa* ATCC 27853. The genes represented in red (up-regulated) and blue (down-regulated) were DEGs with > 1.5-fold changes and a *p*-value ≤ 0.01, while gray dots showed genes with no significant difference compared to the control.

**FIGURE 4:**
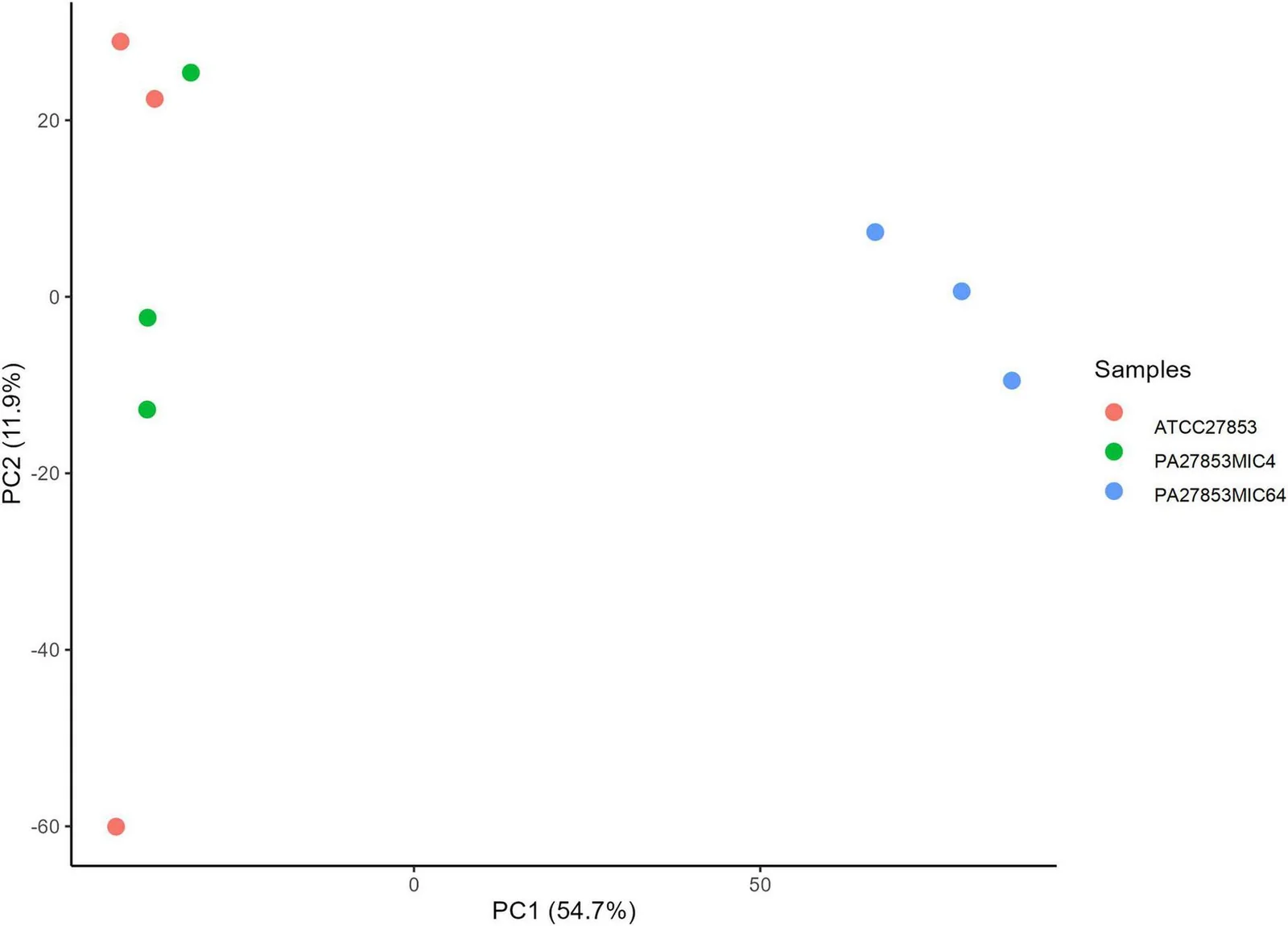
Bi-plot of the principal-component analysis of DESeq2 normalized read counts (all coding genes) for ATCC27853 (red), PA27853MIC4 (green), PA27853MIC64 (blue), split into technical replicates.

WGS analysis showed that PA27853MIC4, PA27853MIC64, and the parental *P. aeruginosa* ATCC 27853 were homologous. Notably, the gene ACG06_03720, encoding a substrate-binding protein of an amino acid ABC transporter, harbored a nonsense mutation (stop codon) in PA27853MIC4 and a further frameshift mutation in PA27853MIC64 (Supplementary Table S1). However, ABC transporters were generally important in antibiotic resistance (Dong et al., 2024). Mechanism of FDC was closely related to bacterial uptake and metabolic systems. To investigate the molecular mechanisms underlying the reduced susceptibility to FDC, we performed RNA-seq on the parental strain ATCC27853 and the induced strains PA27853MIC4 and PA27853MIC64 (Figure 3 and Supplementary Figure S1). RNA-seq data were processed and analyzed for differentially expressed genes (DEGs) using the RUVSeq package, with detailed filtering criteria. RNA-seq analysis revealed that the impact of the FDC on the transcriptome of *P. aeruginosa* ATCC 27853 was time-dependent. The PA27853MIC4 strain had only 50 genes that were changed, with 20 upregulated (40.00%; *p* < 0.05) and 30 downregulated (60.00%; *p* < 0.05) genes. The PA27853MIC64 strain had 2,119 genes with changes in expression, with 1,138 upregulated (53.7%; *p* < 0.05) and 981 downregulated (46.3%; *p* < 0.05) genes. Comparative analysis revealed that higher numbers of DEGs with longer induction times, indicating that the response and regulation under FDC pressure became more active in *P. aeruginosa*. In addition, Figure 4 showed the bi-plot of the principal-component analysis of DESeq2 normalized read counts (all coding genes) for ATCC27853 (red), PA27853MIC4 (green), PA27853MIC64 (blue), split into technical replicates. Principal component analysis (PCA) confirmed that the effect of FDC on *P. aeruginosa* differed significantly relative to *P. aeruginosa* ATCC 27853.

#### 3.3.2 Dynamic correlation of gene expression changes and MIC values

**FIGURE 5:**
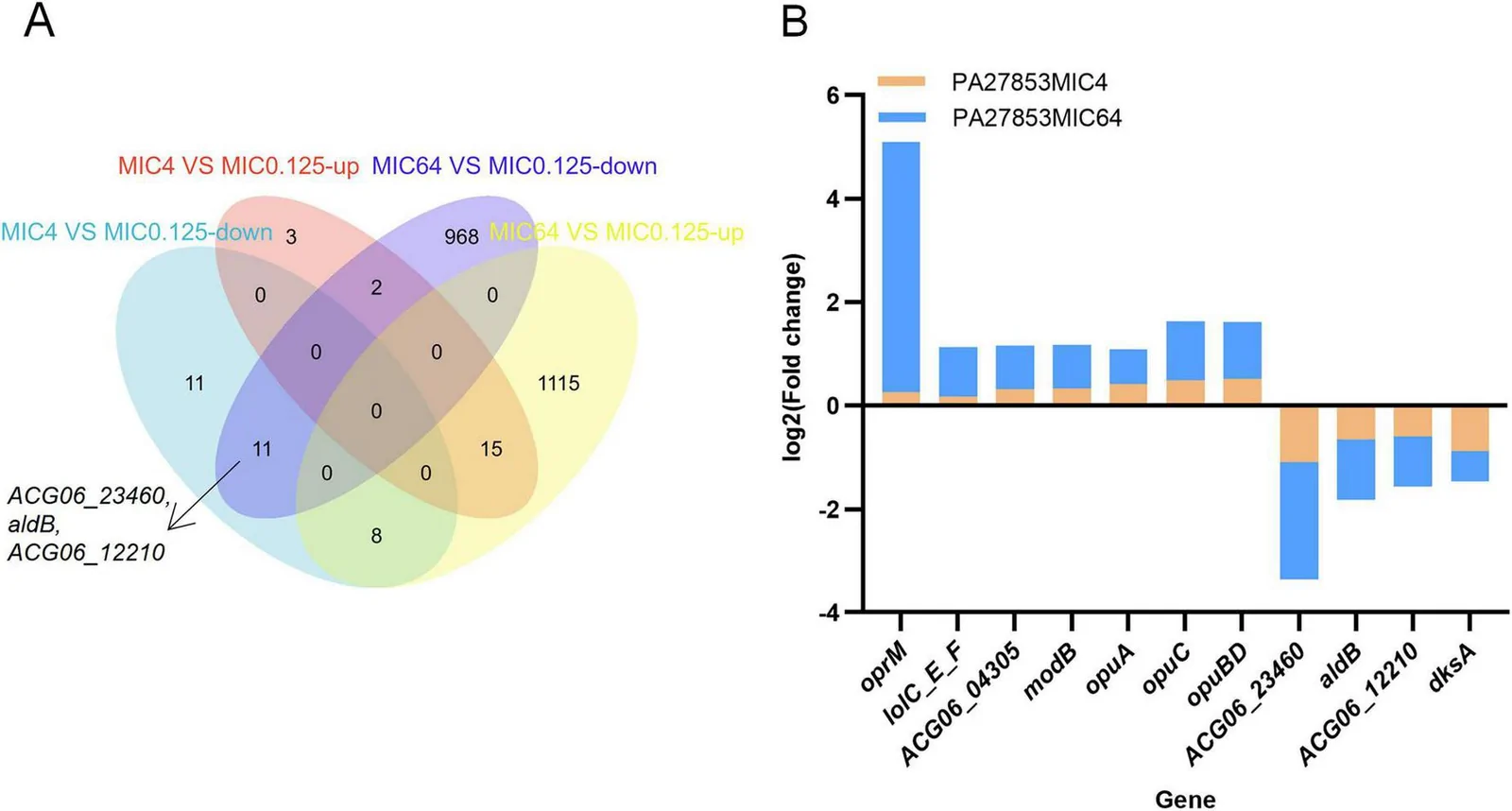
Correlation analysis of gene expression changes and MIC values. **(A)** Venn diagram showing the overlap between up-regulated and down-regulated genes between PA27853MIC4 vs. ATCC27853 and PA27853MIC64 vs. ATCC27853. Among the “common differential genes,” *ACG06_23460, aldB*, and *ACG06_12210* were related to continuously down-regulated genes with MIC. **(B)** The variation of the expression level (log2FC) of key genes with MIC value (4, 64 μg/mL). The orange barplots indicate the expression of PA27853MIC4 genes. The blue barplots indicate the expression of PA27853MIC64 genes.

We analyzed the correlation between the magnitude of gene expression changes (log2FC) and MIC values to elucidate the molecular basis of gradually enhancing resistance. Based on the different expression patterns of genes at MIC = 4 μg/mL and MIC = 64 μg/mL stages, they could be classified into three categories (Figure 5 and Table 1).

**TABLE 1:** Changes in DEGs in PA27853MIC4 and PA27853MIC64 compared with ATCC 27853.

Gene	Products	MIC4		MIC64	
		log2 (fold change)	*P*-value	log2 (fold change)	*P*-value
oprM	RND transporter	0.26	0.036	4.84	0.001
lolC_E_F	Multidrug ABC transporter substrate-binding protein	0.17	0.041	0.96	0.001
ACG06_04305	Methionine ABC transporter ATP-binding protein	0.31	0.049	0.85	0.006
modB	Molybdenum ABC transporter permease	0.33	0.016	0.84	0.003
opuA	Glycine/betaine ABC transporter ATP-binding protein	0.41	0.001	0.68	0.011
opuC	Glycine/betaine ABC transporter substrate-binding protein	0.49	0.001	1.15	0.001
opuBD	Glycine/betaine ABC transporter permease	0.52	0.001	1.09	0.003
ACG06_23460	Conjugal transfer protein	–1.09	0.001	–2.27	0.001
aldB	Aldehyde dehydrogenase	–0.66	0.001	–1.16	0.001
ACG06_12210	Transcriptional regulator	–0.59	0.001	–0.99	0.001
dksA	Molecular chaperone DnaK	–0.88	0.001	–0.58	0.001

##### 3.3.2.1 Continuously up-regulated genes (positively correlated with MIC)

This group of genes was significantly up-regulated at MIC = 4 μg/mL, with a further sharp increase at MIC = 64 μg/mL. Representative genes included the RND efflux pump system-related gene (*oprM*), which showed mild up-regulation at MIC = 4 μg/mL (*log2FC* 0.26) and significant up-regulation at MIC = 64 μg/mL (*log2FC* 4.84). Many ABC efflux pump system-related genes (*lolC_E_F*, *ACG06_04305*, *modB*, *opuA*, *opuC*, *opuBD*) were also up-regulated to varying degrees (log2FC ranged from 0.17 to 0.52) at MIC = 4 μg/mL and (log2FC ranged from 0.65 to 1.15) MIC = 64 μg/mL. This expression pattern aligned closely with the phenotypic leap from 4 to 64 μg/mL, strongly suggesting efflux pumps were a key mechanism to high-level resistance.

##### 3.3.2.2 Continuously down-regulated genes (negatively correlated with MIC)

Genes related to core functions were downregulated or maintained at low expression as the MIC increased. Representative genes included conjugative transfer-related gene (*ACG06_3460*, log2FC -1.09) at MIC = 4 μg/mL, while it continued to down-regulate to -2.27 at MIC = 64 μg/mL. The change in *ACG06_3460* indicated that high concentrations of FDC may severely limit the ability of bacteria to acquire exogenous resistance genes through plasmid exchange, which represents an “energy-saving” strategy under extreme pressure. Some basic metabolic-related genes (*aldB*) showed a continuously decreased (log2FC from -0.66 to -1.16). Transcriptional regulatory-related genes (*ACG06_12210*) continued declined (*log2FCfrom*−0.59*to*−0.98). Although the expression of the protein homeostasis-related gene (*dksA*) did not decrease with MIC, it maintained a low expression in the down-regulation state (log2FC ranged from -0.88 to -0.58). *dksA* severely affects correct folding and repair of proteins. In addition, it can potentially lead to increased intracellular protein stress. Moreover, this makes the bacteria more susceptible to antibiotic clearance (Lu et al., 2023). The downregulation of these genes may reflect an active reduction in growth rate and energy conservation in response to antibiotic pressure, representing a molecular manifestation of fitness cost.

##### 3.3.2.3 Late response genes (threshold response)

Changes in this group of genes were insignificant at MIC = 4 μg/mL and significant at MIC = 64 μg/mL. Representative genes included several ones involved in DNA damage repair (homologous recombination), flagellar assembly, and porphyrin metabolism. This indicated that at the high MIC of 64 μg/mL, the bacteria triggered a broader and deeper global stress, and also remodeling program, which surpassed a single efflux pump mechanism.

#### 3.3.3 Gene ontology enrichment analysis

**FIGURE 6:**
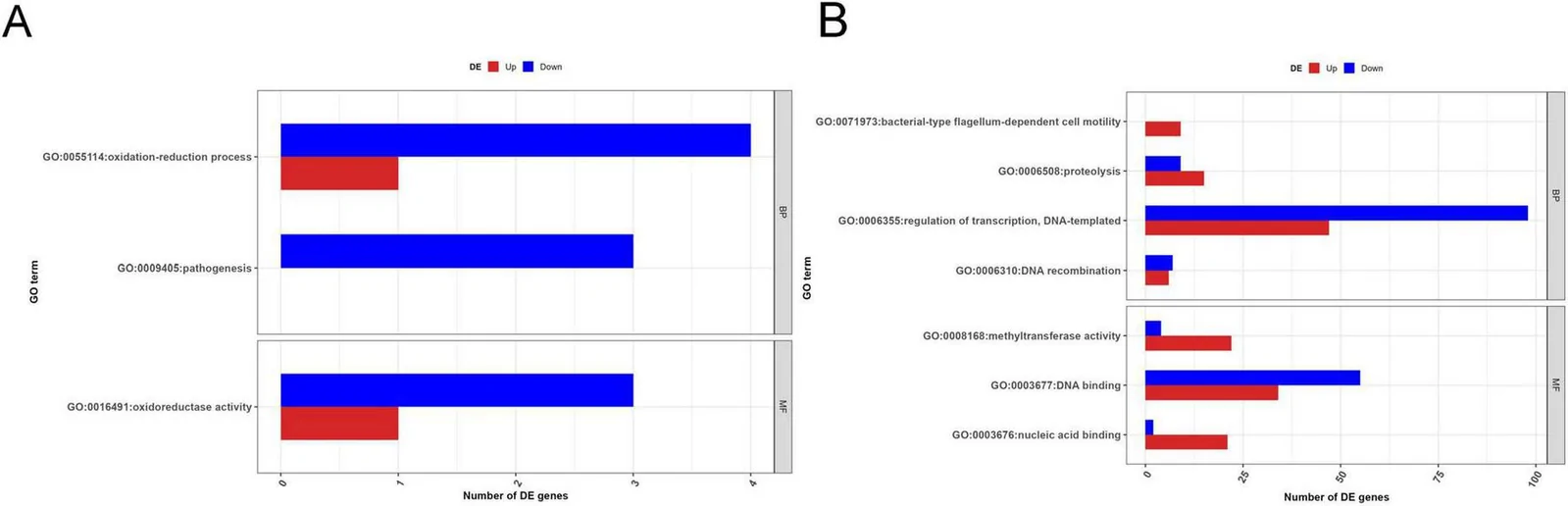
Gene ontology analysis of DEGs. The induced *P. aeruginosa* at different MIC values identified two main categories of biological processes and molecular functions, with different numbers of up-regulated and down-regulated genes at different MIC values. **(A)** Differences in GO enrichment analysis of up-regulated and down-regulated DEGs between PA27853MIC4 and ATCC 27853. **(B)** Differences in GO enrichment analysis of up-regulated and down-regulated DEGs between PA27853MIC64 and ATCC 27853.

GO analysis (Figure 6) of the upregulated genes in the PA27853MIC4 strain indicated that the biological process (BP) was mainly enriched in “oxidation-reduction processes” and the molecular function (MF) was mainly enriched in “oxidoreductase activity.” In contrast, downregulated genes had 10 BP and MF terms. In the BP category, the most enriched terms for the downregulated DEGs were related to “oxidation-reduction processes” and “virulence.” In the MF category, the most enriched terms for the downregulated DEGs were related to “oxidoreductase activity.” Compared with ATCC 27853, the PA27853MIC64 strain yielded 154 BP and MF terms for the upregulated genes. In the BP category, the most enriched terms for UP-regulated DEGs were related to “transcriptional regulation,” “protein degradation,” “bacterial flagellum-dependent cell motility,” and “DNA recombination.” In the MF category, the most enriched terms for up-regulated DEGs were related to “DNA binding,” “nucleic acid binding,” and “methyltransferase activity.” Downregulated genes contained 175 BP and MF entries. The most enriched BP GO terms for down-regulated DEGs were “transcriptional regulation,” “protein degradation,” “DNA recombination,” etc. The most enriched MF GO terms for the downregulated DEGs were “DNA binding,” “nucleic acid binding,” and “methyltransferase activity.”

#### 3.3.4 KEGG pathway enrichment analysis

**FIGURE 7:**
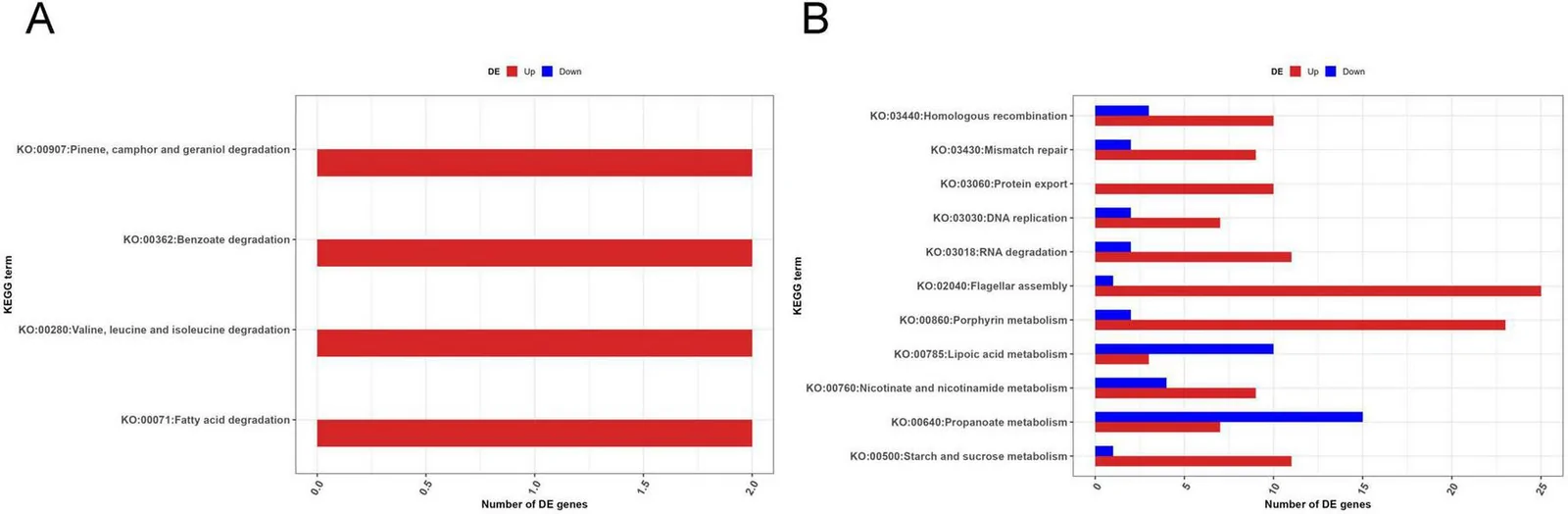
KEGG pathway analysis of DEGs. **(A)** KEGG pathway analysis of up-regulated and down-regulated DEGs between PA27853MIC4 and ATCC 27853. **(B)** KEGG pathway analysis of up-regulated and down-regulated DEGs between PA27853MIC64 and ATCC 27853.

KEGG pathway enrichment analysis of DEGs (Figure 7) revealed that compared to *P. aeruginosa* ATCC 27853, the upregulated genes in the PA27853MIC4 strain were involved in four pathways enriched in “pinene, camphor and geraniol degradation,” “fatty acid degradation,” “benzoate degradation,” and “valine, leucine and isoleucine degradation.” No significant enrichment was observed for downregulated genes. Compared to ATCC 27853, the PA27853MIC64 strain yielded 11 pathways for up-regulated genes and 10 pathways for down-regulated genes, with up-regulated pathways mainly enriched in “flagellar assembly,” “porphyrin metabolism,” “starch and sucrose metabolism,” “RNA degradation,” “homologous recombination,” and “protein export,” while down-regulated pathways were mainly concentrated in “propanoate metabolism” and “lipoic acid metabolism.”

### 3.4 Efflux pump systems were progressively upregulated during the development of cefiderocol (FDC) resistance

To independently validate the transcriptomic findings, we performed RT-qPCR on six key genes identified from the RNA-seq analysis: *oprM, lolC_E_F, modB, opuA, opuC*, and *opuBD*. These genes represent the RND efflux pump system (*oprM*) and multiple ABC transporter systems, all of which exhibited a dynamic upregulation pattern positively correlated with MIC values in the transcriptome data.

The RT-qPCR results (Figrue 8) confirmed the overall transcriptional trend observed by RNA-seq. All six genes displayed higher relative expression levels in the high-level resistant strain PA27853MIC64 compared to the low-level resistant strain PA27853MIC4, supporting the conclusion that efflux pump systems are progressively upregulated during the development of FDC resistance. However, only *oprM, opuC*, and *opuBD* showed statistically significant differential expression (*p* < 0.05). In contrast, *lolCE_F*, *modB*, and *opuA* did not reach statistical significance (*p* > 0.05), but showed an upregulation trend consistent with the RNA-seq data.

**FIGURE 8:**
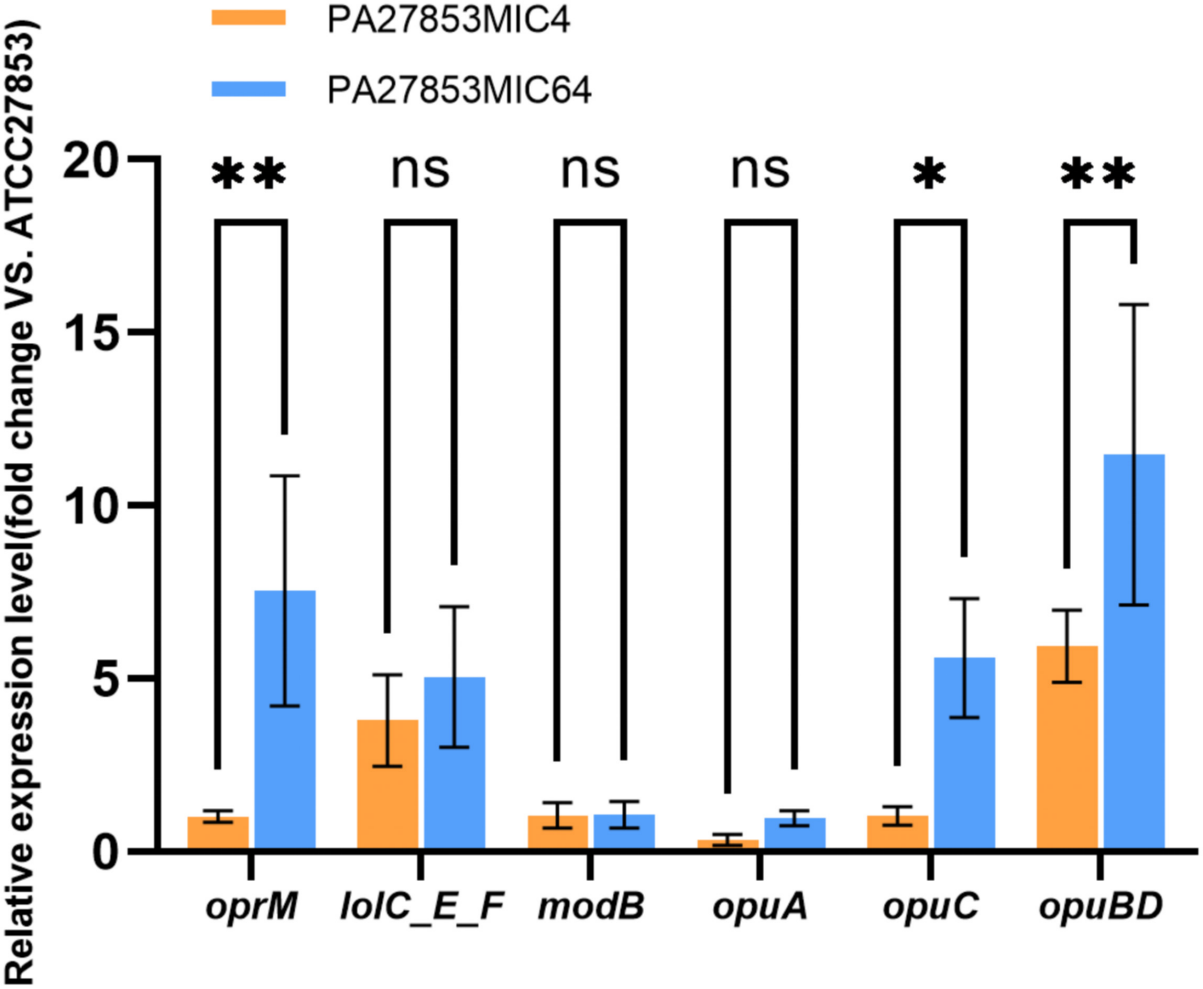
Relative expression levels of six resistance genes in PA27853MIC4 and PA27853MIC64 groups. Take the parental strain ATCC 27853 as the reference. *P < 0.05; **P < 0.001; ns, not significant.

### 3.5 Effects of efflux pump inhibitor PAβN on MIC values

We used PAβN as an inhibitor to confirm that the action of the efflux pumps that conferred FDC resistance to *P. aeruginosa*. In the presence of 25 μg/mL PAβN, MICs for FDC were significantly reduced for the strains PA27853MIC4 (from 4 to 0.5 μg/mL) and PA27853MIC64 (from 64 to 2 μg/mL), as shown in Table 2.

**TABLE 2:** FDC susceptibility of *P. aeruginosa* isolates in the presence and absence of PAβN.

Strain	MIC		The fold change of MIC in inhibition	Expression level of oprM (log2FC vs. ATCC 27853)
	^b^FDC (μ g/mL)	^b^FDC+^a,c^PAβ N (μ g/mL)		
ATCC 27853	0.125	0.125	–	0
PA27853MIC4	4	0.5	8	0.26
PA27853MIC64	64	2	32	4.84

^a^The concentration of PAβN was 25 μg/mL.

^b^FDC, cefiderocol;

^c^PAβN, phenyl-arginine β-naphthylamide.

## 4 Discussion

*P. aeruginosa* had developed high resistance to many antibiotics because of a combination of various resistance mechanisms; an important component is multidrug resistance caused by the external drainage pump system (Miryala et al., 2019). A nationwide survey from Spain showed that various resistance-related gene mutations contribute to the resistance mechanisms of *P. aeruginosa* to FDC, with mutations in efflux pump regulators being one such mechanism (Sastre-Femenia et al., 2025). Our study systematically analyzed the phenotypic changes and molecular mechanisms of *P. aeruginosa* during the evolution of resistance to FDC, revealing a decisive role for efflux pumps and the accompanying physiological remodeling.

In the MIC variation and fitness cost experiments, the resistant strains showed slower growth in the absence of antibiotics. A relative fitness value < 1 indicated a defect in gaining resistance to FDC. Previous studies have indicated that bacteria can develop antibiotic resistance (Wu et al., 2025). This process of inducing resistance involves gene mutations and may be associated with the bacterial growth state and metabolic pathways (Fuzi, 2025; Laborda et al., 2022). For instance, in iron-deficient environments, the growth of the strains is affected because iron is not only an essential nutrient for *P. aeruginosa* but also a mediator of host cell iron-dependent death (Schalk and Perraud, 2023; Shao et al., 2025). Accordingly, our fitness cost experiments indicated that the fitness costs associated with specific resistance mutations were not a non-specific burden equally present in any ecosystem but were related to the environment in which the bacteria were located (Laborda et al., 2022).

We performed WGS and identified a mutation in ACG06_03720. This gene primarily affected the functional alteration of an amino acid ABC transporter. However, ABC transporters were of general importance in antibiotic resistance (Dong et al., 2024). So, the molecular response of *P. aeruginosa* to FDC was investigated by RNA-Seq. By correlating the transcriptomes of strains at different MIC levels, we first depicted dynamic gene expression during the gradual evolution of FDC resistance in *P. aeruginosa*. Previous studies have shown that after Serine hydroxamate induced *P. aeruginosa*, (p)ppGpp induces graded transcriptional changes and promotes antibiotic tolerance (Engelhardt et al., 2025). We found that the upgrade of resistance was not a uniform change in all genes but suggested a possible hierarchical response: (1) Early/basic response, (2) Key contributor, and (3) Global remodeling. The early/basic response involved slight upregulation of genes such as MFS family transporters (*ACG06_10315*, *log2FC 0.895*) possibly mediated the initial adaptation to low-level resistance. The MFS family is the largest and most diverse secondary transporters superfamily (Sauve et al., 2023). They do not contribute to antibiotic efflux but contribute to survival under stress (Adamiak et al., 2024). The Key contributor involved the expression levels of *oprM* and ABC transporter genes, which increased exponentially in the PA27853MIC64 strain, directly correlating with the emergence of high-resistance phenotypes. Specifically, the MexAB-OprM operon is considered the main contributor to antibiotic resistance (López et al., 2017) and shows the largest spectrum of antibiotic substrates (Mazza et al., 2025). Overexpression of this efflux pump is associated with resistance to most antipseudomonal antibiotics (Zahedi Bialvaei et al., 2021). *MexR* is a transcriptional repressor of the mexA-mexB-oprM efflux pump operon (Choudhury et al., 2016). A previous study reported that the MIC of FDC was influenced by the upregulation of the MexAB-OprM multidrug efflux pump, which occurred as a result of mutations in the *mexR* or *nalD* regulatory genes in *P. aeruginosa* (Sadek et al., 2023). In addition, FDC may be a substrate of the MexAB-OprM efflux pump (Ito et al., 2017). FDC/xeruborbactam was a novel β-lactam/β-lactamase inhibitor combination, which retained activity against most *P. aeruginosa* mutants with chromosomally encoded resistance mechanisms. However, the MIC of *P. aeruginosa* PAO1 efflux mutants increased in the xeruborbac tam-based combinations, partly because of constitutive MexAB-OprM efflux (González-Pinto et al., 2025). ABC transporters are involved in the secretion of antibiotics through the cell membrane and contribute to self-resistance to produced antibiotics (Méndez and Salas, 2001). This was supported by the experiment that the efflux pump inhibitor PAβN could significantly decrease the MIC value. Global remodeling: When MIC reached a high threshold (64 μg/mL), the bacteria initiated a large-scale transcriptional reprogramming, involving DNA repair, energy metabolism reprogramming, and motility changes. This was not merely resistance but also a comprehensive evolutionary strategy for maintaining survival and potential adaptability under extreme pressure (Diaz-Diaz et al., 2024). Future studies with time-series transcriptomics were required to validate this dynamic process.

The strategy evolved from PA27853MIC4 to PA27853MIC64. It shifted from a primary and consumptive approach to an advanced and aggressive one. This was similar to the induction of *P. aeruginosa* by Serine hydroxamate (SHX) (Engelhardt et al., 2025). When the MIC was 64 μg/mL, both “DNA recombination” in GO enrichment analyses and “homologous recombination” in KEGG pathways analysis were strongly upregulated simultaneously. The upregulation of homologous recombination pathways prompted us to examine the SOS master regulator RecA. Indeed, RT-qPCR showed a significant increase in RecA expression (Supplementary Figure S2), suggesting a potential genotoxic stress response, although direct measurement of DNA lesions (e.g., comet assay) would be required for definitive confirmation. It activates the strain SOS emergency response to enhance the stress resistance and integrity of the bacteria (Wang M. et al., 2018). During this process, the toxicity also could potentially increase, possibly because efflux pump mutations increase the virulence of *P. aeruginosa* (Fernandes et al., 2025). These two extremes were chosen to contrast the transcriptional differences between a “critical mutation” and a “terminal steady state.” However, a limitation was the lack of intermediate gradients.

Transcriptomic analysis revealed that efflux pumps correlated with the resistance induced by FDC in *P. aeruginosa* ATCC 27853. This is because environmental exposure is closely related to the efflux pump-mediated resistance mechanisms in *P. aeruginosa* (Xu et al., 2020). While our data strongly indicated that transcriptional upregulation of efflux pumps correlated with the elevated cefiderocol MICs, we acknowledged that these findings were associative. Definitive demonstration of a causal role would require future functional validations, such as gene knockout/complementation and direct intracellular drug accumulation assays. The RND excretion pump system, particularly the upregulation of *oprM*, was a key and statistically robust mechanism driving high-level FDC resistance (Lorusso et al., 2022). Moreover, this result suggests that the contribution of ABC transporters is not uniform. In this specific evolutionary lineage, *opuC* and *opuBD* play a more significant role than *lolC_E_F, modB*, and *opuA*. PAβN, as the most well-studied inhibitor of RND efflux systems, binded to the pump and impaired the extrusion of antibiotics, thereby diminishing efflux-mediated resistance (Contreras-Gómez et al., 2022). As shown in Table 2, the MIC had decreased significantly but not drop to the same level as the parental strain after adding PAβN, indicated that efflux pump was one of the important drug resistance mechanisms, but other mechanisms were likely involved. The drug-resistant strains identified in our transcriptome jointly responded to the pressure of antibiotics through complex genetic networks.

Nevertheless, our study has some limitations. First, we used the standard strain ATCC27853 for *in vitro* induction, although clinical isolates have high heterogeneity and their resistance mechanisms might be more complex. However, FDC has only recently been launched in China, and its resistance rate is extremely low. Consequently, research on related clinical strains is still insufficient, and further verification is needed. Second, our induction experiments were conducted in a laboratory, which is different from actual clinical environments, such as host immunity and microbial interactions. These factors may affect the clinical applicability of the results. Future research should focus on inducing drug resistance in different clinical isolates to assess whether the resistance mechanisms vary from strain to strain. Third, this study relied mainly on transcriptomic analysis and did not functionally validate core genes (such as *oprM*) through gene knockout or complementation experiments. Given the multi-gene, multi-level cooperative nature of the resistance network observed here, a single gene knockout may not fully capture the system’s complexity; nonetheless, such experiments are essential to establish causality. This will also become the primary direction of our future research. Fourth, we did not evaluate the fitness cost or pathogenicity changes of the induced resistant strains in animal infection models. Therefore, it was unclear whether a fitness cost was observed *in vitro* or *in vivo*. Finally, the use of only two resistant variants (MIC4 and MIC64) limits our ability to delineate the temporal trajectory of transcriptional changes. Therefore, the proposed “hierarchical model” remains a hypothesis that requires validation through longitudinal time-course transcriptomic analysis during the stepwise selection of FDC resistance. Additionally, the fitness cost comparison between PA27853MIC4 and the wild-type was not performed in this study, precluding a dose-dependent cost evaluation. Future studies should focus on inducing resistance in diverse clinical isolates of *Pseudomonas aeruginosa*, especially those from regions where FDC is increasingly used. This will help assess whether resistance mechanisms vary across strains and provide insights into real-world resistance evolution. Comparative genomic and transcriptomic analyses between standard and clinical strains could reveal strain-specific adaptive pathways. These directions aim to address the current limitations while strengthening the translational relevance of the findings for clinical practice and antimicrobial stewardship.

It was important to note that this study was conducted using a single laboratory-adapted P. aeruginosa strain. Therefore, the observed evolutionary trajectories and transcriptional responses should be considered hypothesis-generating rather than definitive. The extent to which these mechanisms are recapitulated in diverse clinical isolates or under different genetic backgrounds remains to be investigated. Consequently, any potential clinical implications regarding antimicrobial stewardship or combination therapy strategies derived from these data are preliminary and warrant cautious interpretation.

## 5 Conclusion

The evolution of the resistance of *P. aeruginosa* to FDC is associated with the dose-dependent upregulation of core efflux pumps, complemented by stage-specific global physiological remodeling. Targeting this multistage dynamic resistance mechanism with a combination therapy (such as efflux pump inhibitors) or interventions targeting specific stress pathways may be more promising than single antibiotic treatments.

## Data Availability

The datasets presented in this study can be found in online repositories. The names of the repository/repositories and accession number(s) can be found in the article/Supplementary material.

## Footnotes

1
https://www.genome.jp/kegg/

## Supplementary material

Supplementary Figure S1 Clustered heatmap of all DEGs ( > 1.5 fold changes and *p*-value ≤ 0.05) in *P. aeruginosa* across FDC treatment and control. Each column represents one sample, and each row represents one gene. The orange and blue gradients indicate up- and down-regulated gene expression, respectively. A represented PA27853MIC4 VS ATCC27853.B represented PA27853MIC64 VS ATCC27853.

Supplementary Figure S2 Relative expression levels of oprM, mexA, mexB and recA in PA27853MIC4 and PA27853MIC64 groups. Take the parental strain ATCC 27853 as the reference. **P* < 0.05; ***P* < 0.001.
